# Multimodal endovascular management of traumatic carotid-cavernous fistulas: case series and lessons learned

**DOI:** 10.3389/fphar.2025.1602971

**Published:** 2025-05-30

**Authors:** Shangdi Yang, Richao Chen, Mingjia Yi, Chuangnan Li

**Affiliations:** Neurosurgery Department, Jiangmen Hospital of Traditional Chinese Medicine Affiliated to Jinan University, Jiangmen, China

**Keywords:** traumatic carotid-cavernous fistula, endovascular treatment, coil embolization, stent-assisted embolization, multimodal management

## Abstract

**Introduction:**

Traumatic carotid-cavernous fistulas (CCFs) are rare but potentially life-threatening vascular anomalies resulting from head trauma. Oxidative stress markedly disrupts resolution and vascular function, significantly hindering recovery.

**Methods:**

This study presents a case series of six patients diagnosed with CCFs following traumatic events. The cases highlight the complexity of clinical presentation, imaging findings, and treatment approaches, emphasizing the role of advanced endovascular techniques in managing these conditions.

**Results:**

Patients underwent a combination of coil embolization, stent-assisted embolization, and balloon-assisted occlusion, demonstrating the necessity of personalized multimodal treatment strategies. Postoperative outcomes varied, with most patients achieving fistula closure and symptom stabilization, although visual impairments persisted in some cases.

**Conclusion:**

This study highlights the importance of prompt diagnosis, interdisciplinary collaboration, and personalized evolving endovascular interventions in optimizing patient outcomes in traumatic CCFs.

## 1 Introduction

Carotid-cavernous fistulas (CCFs) represent an abnormal arteriovenous connection between the internal carotid artery (ICA) and the cavernous sinus, typically resulting from trauma or vascular anomalies (Al-Shalchy et al., 2024; Alatzides et al., 2023; Henderson and Miller, 2017). While spontaneous CCFs are often linked to connective tissue disorders or congenital malformations, traumatic CCFs frequently arise from high-impact injuries, such as motor vehicle accidents, falls, or penetrating trauma (Texakalidis et al., 2021). These fistulas disrupt normal cerebral hemodynamics, leading to progressive ophthalmic and neurological complications, including proptosis, conjunctival chemosis, and cranial nerve deficits (Henderson and Miller, 2017). Secondary oxidative stress and inflammation, if not properly addressed, can lead to impaired resolution, contributing to long-term morbidity and disability following traumatic brain injury. These processes—encompassing oxidative stress, metabolic dysfunction, inflammation, and excitotoxicity—are mediated at the cellular level, often beyond the resolution of conventional imaging. Nevertheless, they are believed to play a significant role in the progression of secondary injury. In severe cases, these microscopic dysfunctions may manifest macroscopically as diffuse cerebral hyperemia, cytotoxic and/or vasogenic edema, and tissue ischemia, further exacerbating neurological outcomes (Hu et al., 2022).

The classification of CCFs has been traditionally based on their etiology (traumatic vs spontaneous), hemodynamic properties (high-flow vs low-flow), and angiographic features (direct vs. indirect) (Barrow et al., 1985; Zhang et al., 2022). Direct CCFs, often associated with trauma, involve a direct communication between the ICA and the cavernous sinus, whereas indirect CCFs typically involve dural branches of the external or internal carotid artery (Korkmazer et al., 2013). Given the variable presentation of these lesions, early and accurate diagnosis is crucial. Digital Subtraction Angiography (DSA) remains the gold standard for identifying the fistula site, assessing hemodynamic flow, and planning intervention (Timol et al., 2018).

Advancements in endovascular techniques have significantly improved the management of CCFs, offering less invasive alternatives to open surgery (Korkmazer et al., 2013). Coil embolization, stent-assisted embolization, and balloon-assisted occlusion are among the most effective approaches for achieving fistula closure while preserving normal vascular function (Markoutsas et al., 2024). In select cases, a transvenous approach via the superior ophthalmic vein or inferior petrosal sinus may be required to access challenging fistula sites (Hong Duc et al., 2020; Thiex et al., 2014). Despite these advancements, treatment challenges persist, particularly in cases with complex vascular anatomy, incomplete occlusion, or recurrence following initial intervention.

This paper presents six cases of traumatic CCFs treated with multimodal endovascular approaches, discussing diagnostic challenges, procedural intricacies, and postoperative outcomes. Through this case series, we aim to highlight the importance of individualized treatment strategies, interdisciplinary collaboration, and ongoing innovation in neurovascular intervention.

## 2 Methods

### 2.1 Patients

This study included six patients diagnosed with traumatic CCFs at our institution. The patients ranged in age from 18 to 64 years and presented with varying degrees of ophthalmologic and neurological symptoms following traumatic events such as falls or head injuries. Each patient underwent a thorough clinical evaluation, including ophthalmologic and neurological assessments, to determine the extent of vascular involvement.

### 2.2 Imaging analysis

All patients underwent DSA, which remains the gold standard for diagnosing CCFs. DSA provided high-resolution visualization of the arteriovenous communication between the ICA and the cavernous sinus, enabling accurate classification of the fistulas. Additional imaging modalities, including computed tomography angiography (CTA) and magnetic resonance imaging (MRI), were utilized in select cases to assess associated intracranial injuries, venous drainage patterns, and collateral circulation. All patients underwent Digital Subtraction. While DSA is essential for definitive diagnosis, non-invasive imaging techniques such as transocular ultrasound and Doppler can be valuable adjuncts for bedside pre- and postoperative assessment without radiation exposure, though these were not systematically applied in the present study.

### 2.3 Treatment approaches

Endovascular embolization was the primary treatment modality for all patients. The choice of embolization technique was tailored based on the anatomical characteristics of the fistula, vascular access, and hemodynamic factors. The following treatment strategies were employed:

*Coil Embolization:* In cases where direct access to the fistula was feasible, detachable coils were used to occlude the arteriovenous shunt while preserving normal vascular structures.

*Stent-Assisted Embolization:* In select cases with high-flow fistulas, a stent was deployed to provide structural support and prevent coil migration.

*Balloon-Assisted Occlusion:* Balloon catheters were used in conjunction with coil embolization to achieve controlled occlusion of the fistula.

*Glue Embolization:* Liquid embolic agents, such as Onyx-18, were used in cases where residual shunting was present despite coil embolization.

The transarterial approach was the preferred route for embolization, with the femoral artery serving as the primary access site. In cases with challenging arterial access, a transvenous approach through the superior ophthalmic vein or inferior petrosal sinus was used.

### 2.4 Postoperative monitoring and follow-up

Patients were closely monitored in the post-procedural period for neurological status, ocular symptoms, and potential complications. Follow-up DSA was performed in all cases to assess the durability of embolization and detect any residual or recurrent shunting. Patients with persistent visual deficits or residual fistula flow were scheduled for additional interventions as needed.

## 3 Results

### 3.1 Case 1. management of traumatic left carotid-cavernous fistula with two-stage endovascular intervention in a 38-year-Old male

**FIGURE 1:**
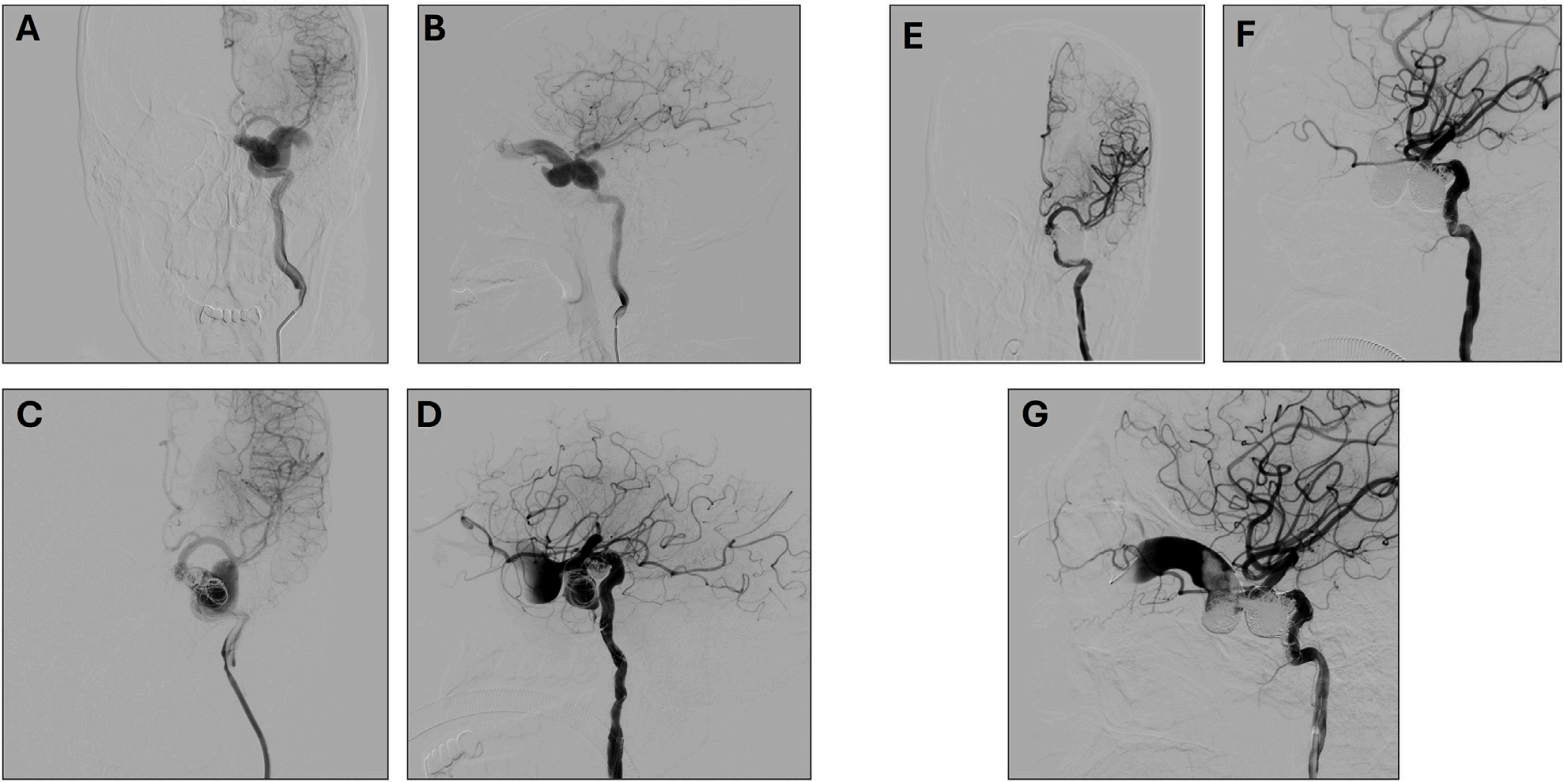
**(A, B)** Preoperative Left Internal Carotid Artery Anteroposterior **(A)** and lateral **(B)** angiography. **(C, D)** First Postoperative Left Internal Carotid Artery Anteroposterior **(C)** and lateral **(D)** angiography. **(E, F)**. Second post-operative left Internal Carotid Artery Anteroposterior **(E)** and lateral **(F)** angiography **(G)** Ophthalmic Venous Access Imaging.

A 38-year-old male presented with mild edema in the left eyelid, along with slight exophthalmos, mild ptosis, and mild conjunctival congestion on physical examination. No abnormalities were observed in ocular movements, and no scleral icterus was present. Lens opacities were noted. The left eye was blind, while the right eye exhibited normal vision. The left pupil had a diameter of approximately 4 mm, with an absent light reflex. The right pupil had a diameter of about 3 mm, with a normal light reflex. A blowing sound synchronous with the pulse was detected upon auscultation of the left eye. DSA performed on 19 June 2018, revealed a left ICA cavernous sinus fistula, associated with significant venous congestion in the affected eye. However, the precise location of the fistula could not be definitively determined based on the imaging findings. Figures 1A, B shows preoperative left ICA anteroposterior angiography.

A first surgery was performed on 22 June 2018, under general anesthesia. The Seldinger technique was used to puncture the right femoral artery, and an 8F sheath was successfully inserted. A 6F catheter was advanced to perform left ICA angiography, confirming the presence of a cavernous sinus arteriovenous (AV) fistula. The 6F Envoy guiding catheter was advanced to the cervical segment of the left ICA, and a microcatheter was employed to precisely identify the fistula’s location. To occlude the fistula, a series of embolization coils (14 × 30, 12 × 30, 10 × 30, 9 × 30) were deployed.

During the filling process, while adjusting the 5 × 10 coil, it unwound, with portions prolapsing through the fistula opening into the ICA. To mitigate this, a balloon was temporarily inflated to compress the distal end of the fistula. Attempts to reposition the prolapsed coils were unsuccessful, as they could not be reinserted into the fistula opening. Given the high risk of additional coil migration into the ICA with continued embolization, a stent was deployed to safeguard the ICA near the fistula. Following the deployment of the stent’s distal end, tirofiban was administered to prevent thrombosis. Subsequently, a 5 × 10 coil was introduced to further occlude the fistula. Upon full stent deployment, the coil was detached, and angiographic assessment confirmed that the prolapsed segment adhered securely to the vessel wall. Post-embolization angiography demonstrated partial occlusion of the fistula with preserved distal ICA perfusion. Considering the challenges of further embolization via the transarterial route, the procedure was concluded. (Figures 1C, D).

A second surgery was performed on 2 August 2018. During this procedure, angiography revealed coil embolization at the fistula site, but the fistula persisted with some retrograde flow to the eye veins. A second intervention via left external jugular vein access was attempted but was unsuccessful. The eye venous approach was then used, allowing successful access and embolization of the remaining fistula (Figures 1E–G). Post-operative evaluation on 14 August 2018, showed that the patient was awake with stable vital signs, left-eye blindness, no significant headache, and normal right-eye vision.

### 3.2 Case 2. endovascular treatment of right carotid-cavernous fistula in a 31-year-Old female following trauma

**FIGURE 2:**
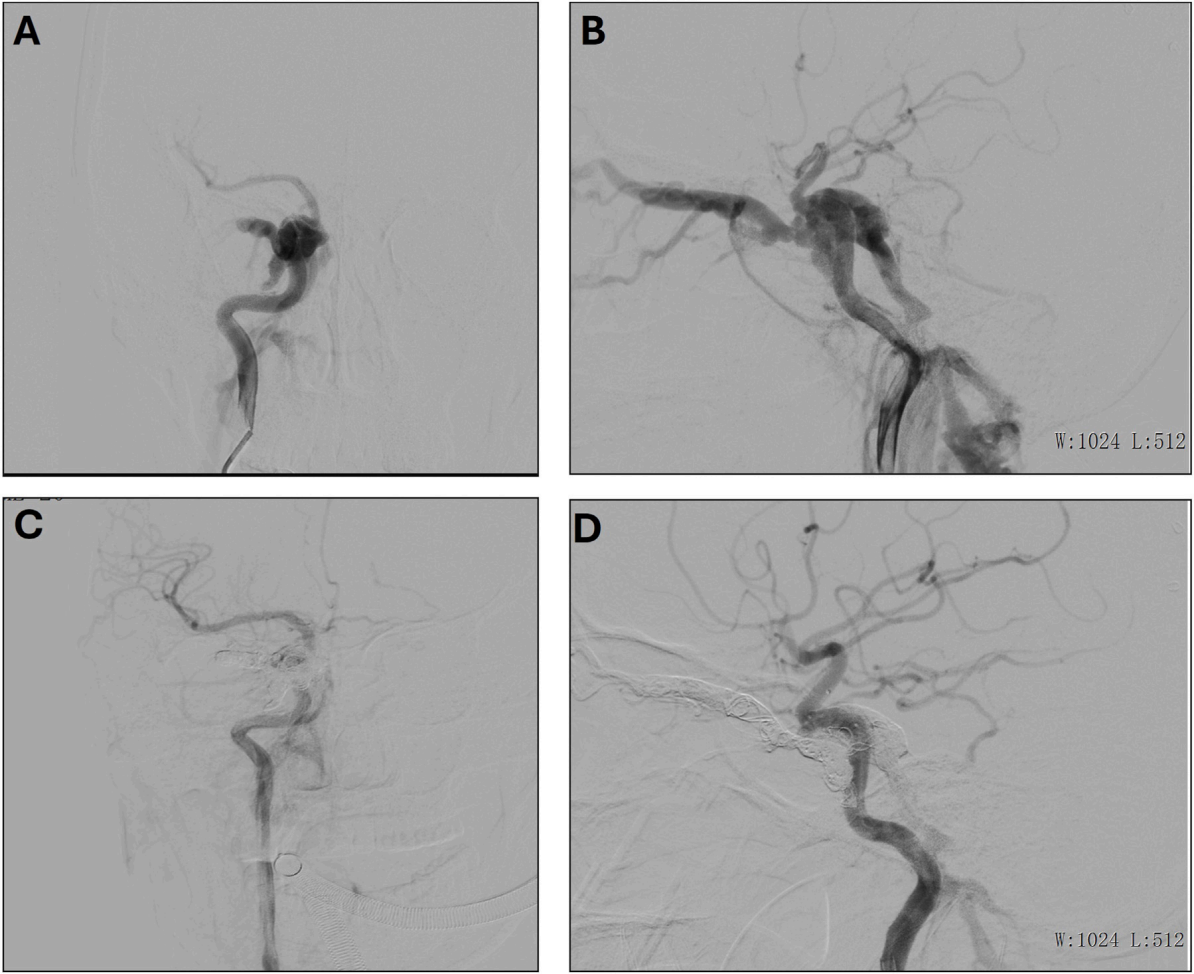
**(A, B)** Preoperative frontal **(A)** and lateral **(B)** imaging of the right internal carotid artery. **(C, D)** Postoperative frontal **(C)** and lateral **(D)** imaging of the right internal carotid artery.

A 31-year-old female presented with a craniocerebral trauma, including brain contusion, subarachnoid hemorrhage, and a right temporal bone fracture. Clinically, she exhibited right eye conjunctival congestion and slight proptosis. The patient also reported mild right ear tinnitus for the past 2 months and right eye ball congestion for the past 2 days. Additionally, hyperuricemia was noted in her medical history. A surgical intervention was performed under general anesthesia. Access to the right femoral artery was obtained via the Seldinger technique, and a 5F catheter was advanced for angiographic imaging. Angiography confirmed the presence of a right ICA cavernous sinus fistula, with arterial flow shunting into the ophthalmic veins and contralateral cavernous sinus (Figures 2A, B). Fistula embolization was attempted using a combination of coils and embolic glue; however, the anatomical complexity of the fistula hindered complete occlusion. Multiple embolization attempts were made, resulting in partial occlusion of the fistula (Figures 2C, D). Post-operatively, the patient experienced resolution of the right ear vascular murmur. Although some residual headaches persisted, the patient’s overall condition improved, and she was closely monitored for ongoing recovery.

### 3.3 Case 3. severe head and chest trauma with bilateral carotid-cavernous fistula in 18-year-Old patient following fall

**FIGURE 3:**
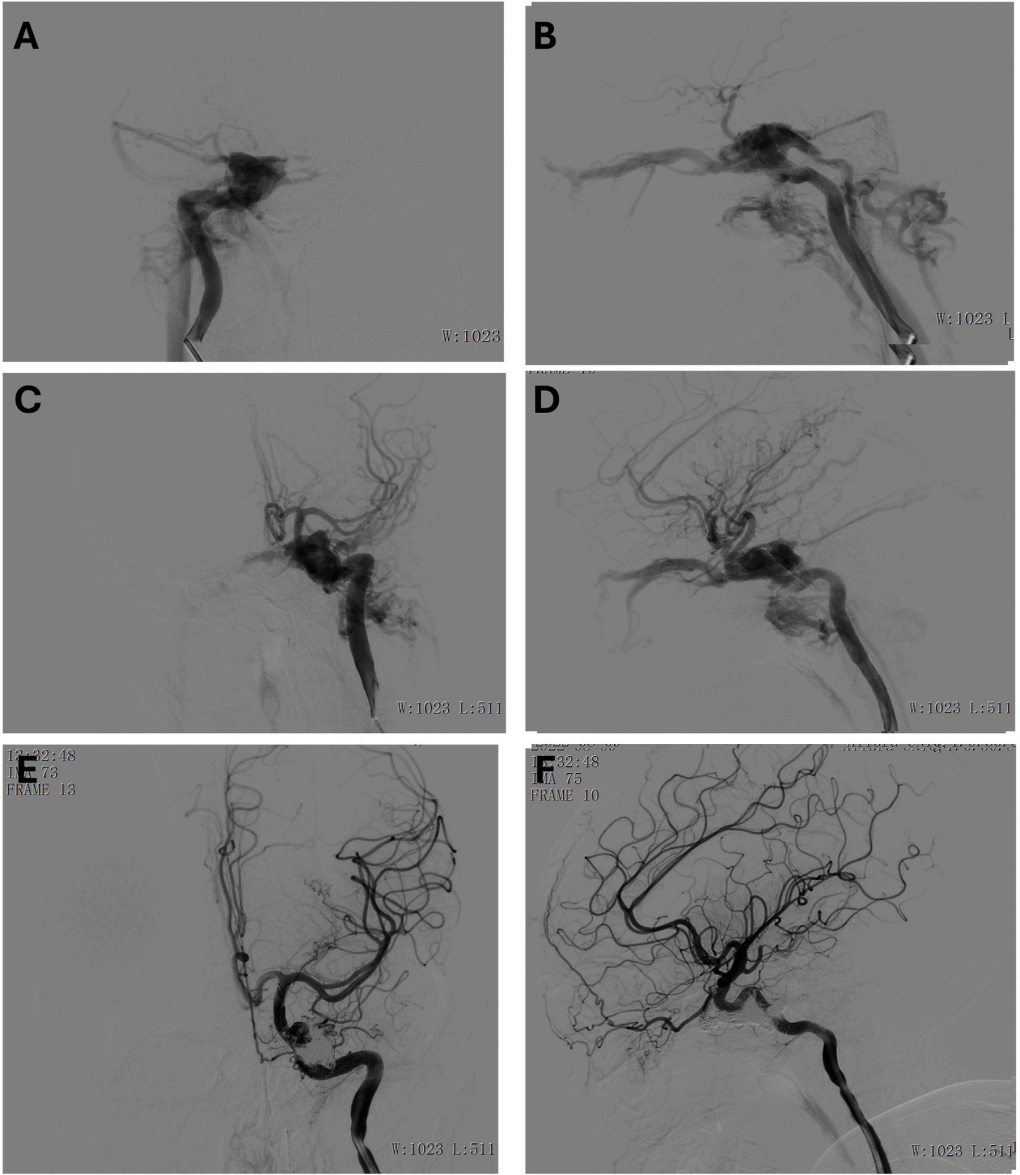
**(A, B)** Preoperative anteroposterior **(A)** and lateral **(B)** view of the right internal carotid artery. **(C, D)** Preoperative anteroposterior **(C)** and lateral **(D)** view of the left internal carotid artery. **(E, F)** Postoperative anteroposterior **(E)** and lateral **(F)** view of the left internal carotid artery.

An 18-year-old male presented with severe trauma following a high-impact injury. Upon arrival, he was in a coma with a Glasgow Coma Scale (GCS) score of 5, indicating severely impaired neurological status. Clinical examination revealed bilateral fixed pupils, and imaging studies suggested the presence of a significant open traumatic brain injury, which included brain herniation, multiple contusions, and a subdural hematoma. In addition to the brain injury, the patient had other associated trauma-related injuries, including pulmonary contusions, rib fractures, and a traumatic eye rupture. A detailed angiographic evaluation under general anesthesia revealed the presence of bilateral internal carotid artery (ICA) cavernous sinus fistulas (CSFs), with significant arterial-to-venous shunting (Figures 3A–D). These vascular injuries contributed to an abnormal hemodynamic state, which compounded the patient’s neurological deterioration.

To address the cavernous sinus fistulas, the interventional radiology team employed a combination of coils and embolic agents to occlude the fistulas, effectively halting the abnormal shunting between the internal carotid arteries and the cavernous sinuses. The procedure was successful, and post-embolization angiography demonstrated a satisfactory outcome (Figures 3E, F), with significant reduction in shunting. The patient was monitored in the intensive care unit, with ongoing management for his multiple injuries. Although he remained in a coma for some time after the procedure, gradual neurological improvement was observed. The timely diagnosis and intervention for the bilateral ICA cavernous sinus fistulas played a critical role in stabilizing his condition and contributing to his recovery.

### 3.4 Case 4. traumatic left carotid-cavernous fistula with left eye blindness in a 64-year-Old female: Successful endovascular treatment

**FIGURE 4:**
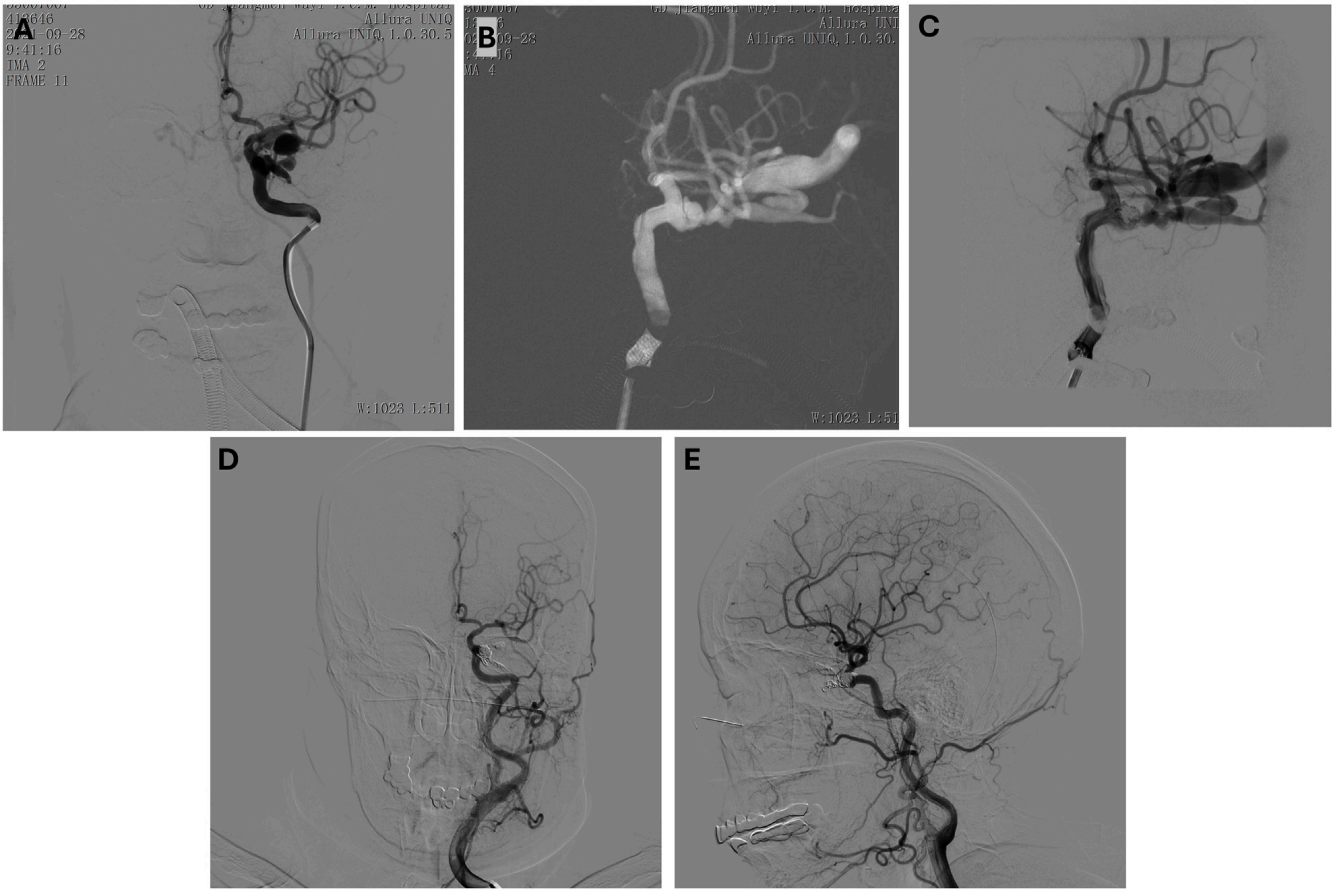
**(A–C)** Preoperative anteroposterior **(A)** and lateral **(B, C)** view of the left internal carotid artery. Microcatheter in position, coil embolization performed. **(D, E)** Post-embolization anteroposterior **(D)** and lateral **(E)** view.

A 64-year-old female presented with symptoms following a traumatic injury, including sudden vision loss in her left eye and signs of ocular and neurological distress resulting in a left internal carotid artery (ICA) cavernous sinus fistula. On examination, she had left eye blindness, and further investigation revealed a traumatic fistula at the left ICA cavernous sinus region, which contributed to significant clinical findings, including left eye blindness. The patient’s condition required immediate intervention to address the fistula and prevent further neurological complications. Angiographic imaging confirmed the presence of the traumatic fistula (Figures 4A–C), revealing abnormal communication between the left ICA and the cavernous sinus, which led to disturbed blood flow and ocular symptoms. A microcatheter was carefully navigated into the affected region to treat the fistula, and a combination of coil embolization and glue injection was employed to occlude the fistula. This procedure successfully achieved a satisfactory closure of the abnormal connection between the artery and the sinus, thereby stabilizing the patient’s hemodynamics and preventing further progression of the fistula (Figures 4D, E). Post-operative follow-up revealed that the patient’s left eye blindness persisted, though there was a slight improvement in her vision impairment. While the overall ocular condition did not fully recover, the intervention successfully addressed the vascular abnormality, preventing further neurological deterioration and stabilizing her condition.

### 3.5 Case 5. ruptured internal carotid artery bifurcation aneurysm with subarachnoid hemorrhage and left carotid-cavernous fistula: Complex endovascular management in a 64-year-Old female

A 64-year-old female presented with acute onset of dizziness and headache lasting for 23 h. Initial clinical assessment in the emergency department suggested a diagnosis of intracranial hemorrhage. Further diagnostic workup revealed multiple comorbidities, including a ruptured aneurysm at the bifurcation of the ICA with concomitant subarachnoid hemorrhage, left ICA cavernous sinus fistula, hydrocephalus, intracranial infection, *Pseudomonas aeruginosa* pneumonia, hypertension grade 3, coronavirus infection, urinary tract infection, hyperammonemia, hypoalbuminemia, and hypokalemia. On admission, the patient was lethargic, exhibiting a reduced level of consciousness but responsive to verbal stimuli. Speech was slurred, and the patient was able to answer questions appropriately, reporting dizziness, headache, blurred vision in the right eye, nausea, and generalized weakness. Neurological examination revealed no signs of limb twitching, opisthotonus, or trismus, and respiratory examination showed no signs of dyspnea or cyanosis. The patient was afebrile, with no signs of incontinence or other autonomic dysfunction.

In the surgical setting, the patient was positioned supine on the operating table, and general anesthesia was successfully induced. After routine aseptic preparation, a modified Seldinger technique was used to puncture the right femoral artery and a 6F vascular sheath was inserted. Systemic anticoagulation was achieved with heparin, and a 6F pig-tail catheter was used to perform an aortic arch angiogram, which revealed a type I aortic arch. A 5F single-curve diagnostic catheter was advanced into the bilateral internal carotid arteries, vertebral arteries, and subclavian arteries. A 3D rotational angiogram of the left ICA demonstrated a wide-necked, saccular aneurysm at the bifurcation of the left ICA, measuring approximately 9.17 mm × 4.27 mm x 5.74 mm. The aneurysm was predominantly supplied by the left ICA, and no significant abnormalities were noted in the remaining vascular structures. Based on these findings, stent-assisted embolization was planned.

An 8F vascular sheath was exchanged, and an 8F guiding catheter was advanced to the distal cervical segment of the left ICA under the guidance of a microguidewire. An intermediate catheter was advanced to the cavernous sinus segment of the left ICA. Using the roadmapping technique, a Headway-21 microcatheter was navigated into the M1 segment of the middle cerebral artery, followed by placement of a Headway-17 microcatheter into the aneurysm. A 7 mm × 22 cm coil was deployed into the aneurysm, and the coil was released. Following intravenous administration of Verapamil, an LVIS 3.5 mm × 20 mm partially deployed stent was placed to provide a scaffolding effect. Another 7 mm × 22 cm coil was deployed, forming a basket-like structure within the aneurysm, followed by the deployment of additional coils (5 mm × 15 cm, 4 mm × 10 cm, 4 mm × 10 cm, 2 mm × 8 cm, 2 mm × 6 cm, 1.5 mm × 4 cm) to ensure complete embolization. Post-embolization angiography confirmed dense embolization of the aneurysm, with no residual flow within the aneurysm sac.

**FIGURE 5:**
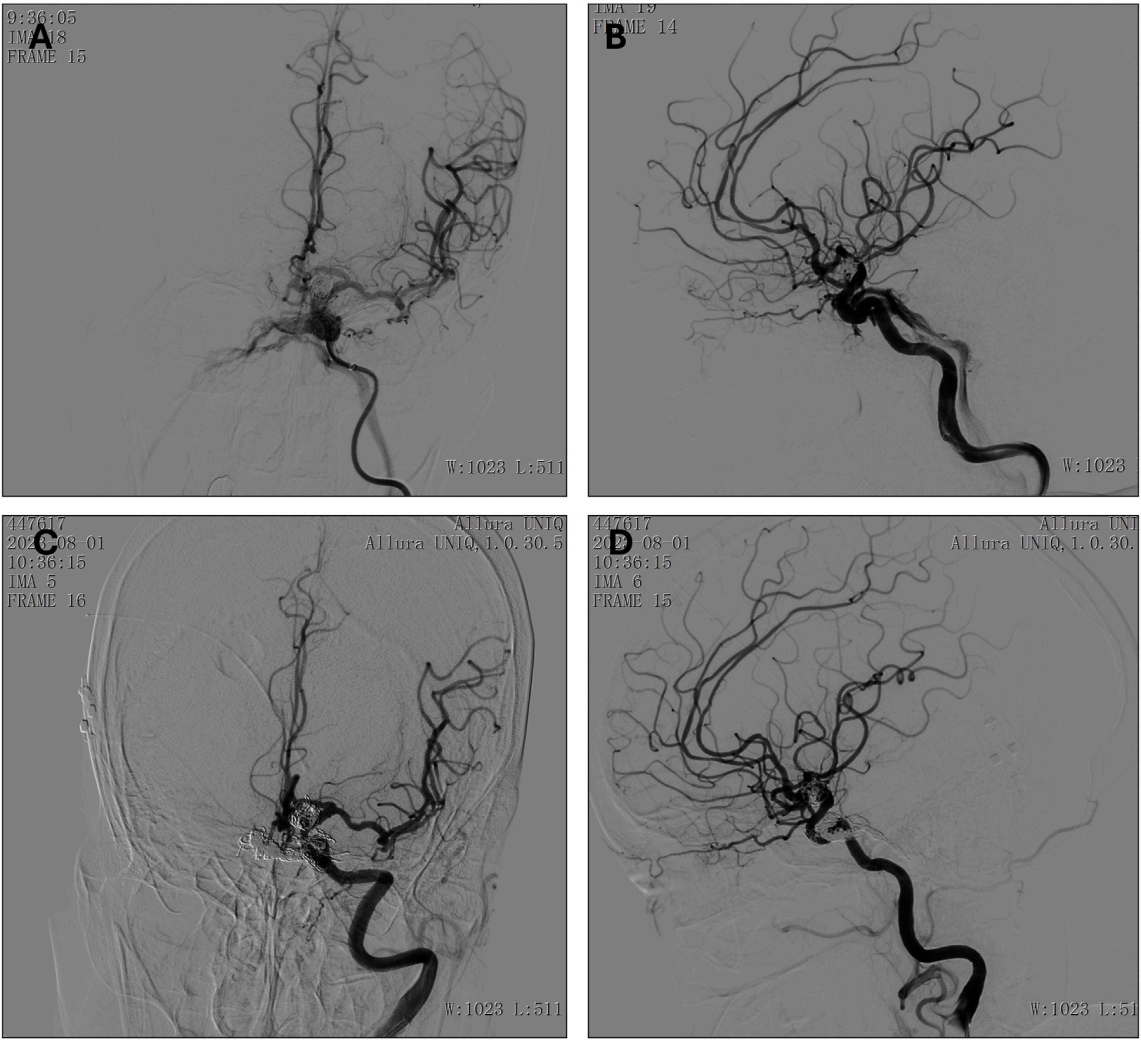
**(A, B)** Preoperative anteroposterior **(A)** and lateral **(B)** view of the left internal carotid artery. **(C, D)** Postoperative (8 months follow-up) anteroposterior **(C)** and lateral **(D)** view of the left internal carotid artery.

Post-procedural angiography using a lateral view of the left ICA confirmed the presence of the cavernous sinus fistula, with the fistula orifice located in the cavernous sinus segment of the left ICA (Figure 5). The fistula was found to drain into the superior and inferior ophthalmic veins, the pterygoid plexus, and the superior and inferior petrosal sinuses. No significant abnormalities were noted in the remaining vascular territories.

A 6F vascular sheath was inserted into the left femoral vein, and the Headway-17 microcatheter was advanced under the guidance of a Traxcess microguidewire through the fistula into the cavernous sinus. A balloon catheter was then positioned in the cavernous sinus orifice of the right ICA. Coils (10 mm × 30 cm, 8 mm × 30 cm, 7 mm × 30 cm, 7 mm × 30 cm, 6 mm × 20 cm, 6 mm × 20 cm) were deployed to embolize the fistula. Following balloon deflation, additional coils (5 mm × 15 cm, 5 mm × 15 cm) were deployed, and intermittent imaging was performed to assess the degree of occlusion. Angiographic results showed a significant reduction in the fistula flow. Additional coils (9 mm × 24 cm, 6 mm × 20 cm, 6 mm × 18 cm, 5 mm × 12 cm) were deployed, followed by the injection of embolic glue under intermittent fluoroscopic guidance. This resulted in further reduction in cavernous sinus opacification, and final angiography showed reduced fistula flow with preservation of distal arterial patency.

### 3.6 Case 6. endovascular treatment of left carotid-cavernous fistula with adjacent orbital and ocular manifestations in a 46-year-Old female

A 46-year-old female presented with right eye redness, pain, and a foreign body sensation for more than 10 days. The outpatient diagnosis considered an inflammatory orbital pseudotumor of the right eye. The final diagnosis included: 1. Left ICA cavernous sinus fistula, 2. Paralytic strabismus in both eyes, 3. Entropion and trichiasis in the right eye, and 4. Keratitis in the right eye. Upon admission, the patient experienced right eye redness, pain, blurred vision, and diplopia but no dizziness, headaches, nausea, or vomiting. Her appetite was acceptable, sleep was disturbed, and her bowel and urinary functions were normal. On examination, her visual acuity was 0.15 in the right eye and 0.3 in the left eye. Intraocular pressure was 27.0 mmHg in the right eye and 19.5 mmHg in the left eye. Both eyes showed limited outward gaze. In primary gaze, the right eye had an internal deviation of approximately 30°, with the lower eyelid showing entropion, where the eyelashes were directed toward the cornea, causing friction and conjunctival hyperemia. A grayish-white infiltration was noted in the inferior cornea. The left cornea appeared clear, and both eyes were negative for keratic precipitates. The anterior chamber depth was normal, the aqueous humor was clear, the iris patterns were distinct, and the pupils were round at 3 mm with a normal light reflex. Mild lens opacities were noted, and fundoscopy was not possible.

For the diagnostic angiography, the patient was placed in the supine position, and after routine disinfection and draping, 2% lidocaine was applied for local anesthesia. The modified Seldinger technique was used to puncture the left femoral artery and place a 5F vascular sheath. After systemic heparinization, a 5F pig-tail catheter was used for aortic arch angiography, which showed a type I aortic arch. A 5F curved catheter was positioned in the bilateral common carotid arteries, internal carotid arteries, external carotid arteries, subclavian arteries, and vertebral arteries for angiography, with imaging of the cervical and intracranial segments. A three-dimensional rotational angiogram of the left ICA revealed a cavernous sinus fistula with the fistula site located on the inferior wall of the cavernous sinus segment, draining into the superior petrosal sinus, superior ophthalmic vein, inferior ophthalmic vein, intercavernous sinus, and pterygoid plexus veins. The anterior communicating artery and left posterior communicating artery were patent, with good collateral circulation. The procedure was completed successfully, and after heparin neutralization with protamine sulfate, the femoral sheath was removed. Hemostasis was achieved by manual compression for 15 min, and sterile dressings were applied. No bleeding was noted at the puncture site, and dorsalis pedis pulses were intact.

**FIGURE 6:**
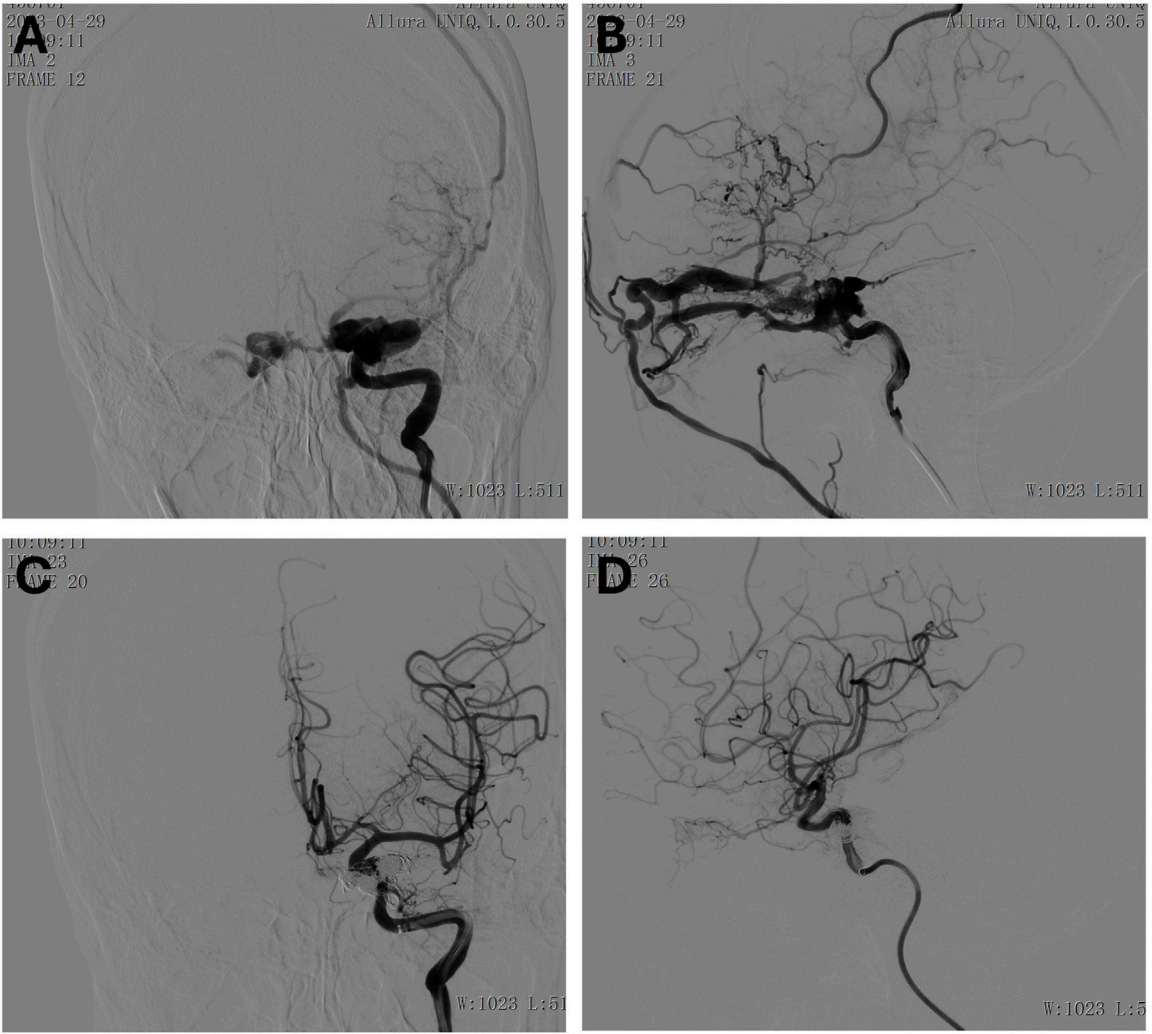
**(A, B)** Preoperative anteroposterior **(A)** and lateral **(B)** view of the left internal carotid artery. **(C, D)** Postoperative anteroposterior **(C)** and lateral **(D)** view of the left internal carotid artery.

For the interventional procedure, the patient was placed supine and underwent general anesthesia with endotracheal intubation. After standard disinfection and draping, a modified Seldinger technique was used to puncture the right femoral artery and place a 6F vascular sheath. The patient was systemically heparinized. A 6F Envoy catheter was placed in the cervical segment of the left internal carotid artery with the assistance of a Glidewire. A Headway-17 microcatheter was advanced through the fistula into the origin of the superior ophthalmic vein, and another Headway-17 microcatheter was navigated through the fistula into the cavernous sinus. A Complex 18 coil (20 mm × 50 cm) was deployed via the superior ophthalmic vein microcatheter. However, during the advancement, there was insufficient support from the guiding catheter, and vessel spasm was noted, possibly due to the tortuosity of the left ICA. An 8F vascular sheath was then placed, and an 8F guiding catheter was used to guide a SilverSnake intermediate catheter to the petrous segment, with the Glidewire assisting in navigation. Two Headway-17 microcatheters were again advanced through the fistula into the cavernous sinus and superior ophthalmic vein. Multiple coils were deployed, including a Complex 18 20 mm × 50 cm, Helical 18 Regular 18 mm × 30 cm, Helical 18 Regular 18 mm × 30 cm, Helical 18 Regular 18 mm × 30 cm, Helical 18 Regular 14 mm × 30 cm, Helical 18 Regular 10 mm × 30 cm, and Helical 18 Regular 6 mm × 20 cm, which successfully occluded the cavernous sinus fistula. After the superior ophthalmic vein microcatheter was withdrawn, a small portion of the fistula still drained into the superior petrosal sinus. A DMSO solution (0.3 mL) was injected through the microcatheter, followed by EVAL-I glue. Sequential angiographic imaging showed a progressive reduction in cavernous sinus filling, and after 0.5 mL of glue was injected, the cavernous sinus fistula was completely occluded. Final angiography confirmed that distal blood flow was preserved (Figure 6). The procedure was successful, and protamine sulfate was used to neutralize heparin. The right femoral sheath was removed, and manual compression was applied for 15 min. The puncture site showed no bleeding, and dorsalis pedis pulses were intact. At discharge, the patient’s condition had improved, with clear vision and resolution of the pain and redness. She no longer experienced double vision, dizziness, headaches, nausea, or vomiting. Her appetite and sleep had normalized, and her bowel and urinary functions were stable. Her pupils were round, with a diameter of about 3 mm and a normal light reflex. The right eye exhibited limited abduction.

## 4 Discussion

The management of traumatic CCFs presents several challenges that necessitate a multidisciplinary approach. The cases examined in this study, summarized in Tables 1, 2, highlight the complex nature of traumatic CCFs and emphasize the importance of individualized treatment strategies. The primary goal of treatment is to restore normal hemodynamics while preserving vascular integrity and preventing neurological deterioration (van Rooij et al., 2006). Oxidative stress is a key driver of injury, which contributes to vascular dysfunction in CCFs, exacerbating endothelial damage and impairing recovery (Hu et al., 2022).

**TABLE 1:** Summary of the cases reported in this study.

Case	Diagnosis	Surgical procedure
Case 1: 38-y.o. Male	Traumatic Left ICA Cavernous Sinus Fistula	Two-stage endovascular intervention with coil embolization and stent placement
Case 2: 31-y.o. Female	Traumatic Right ICA Cavernous Sinus Fistula, Craniocerebral Trauma	Endovascular embolization using coils and embolic glue
Case 3: 18-y.o. Male	Severe Open Traumatic Brain Injury, Bilateral ICA Cavernous Sinus Fistulas	Bilateral coil and embolic glue occlusion of ICA cavernous sinus fistulas
Case 4: 64-y.o. Female	Traumatic Left ICA Cavernous Sinus Fistula, Left Eye Blindness	Microcatheter-based coil embolization and glue injection
Case 5: 64-y.o. Female	Ruptured Left ICA Bifurcation Aneurysm, Left ICA Cavernous Sinus Fistula, Subarachnoid Hemorrhage	Stent-assisted coiling of aneurysm, embolization of cavernous sinus fistula
Case 6: 46-y.o. Female	Left ICA Cavernous Sinus Fistula with Ocular Manifestations	Transvenous embolization using coils and glue through superior ophthalmic vein

**TABLE 2:** Long-term clinical and visual outcomes following endovascular treatment of CCFs.

	Time between the initial procedure and follow-up DSA	Follow-up angiography and individual outcomes	Visual outcomes	Postoperative long term clinical course
Case 1	1 year	1st surgery: contrast agent retention at the CCF site. Preserved distal vessel perfusion 2nd surgery: complete occlusion	PRE: left eye blind. Left pupil 4 mm in diameter, with absent light reflex POST (1 month): left eye blind. Axial length: 22.0 mm, anterior chamber depth: 2.6 mm, lens thickness: 3.7 mm, vitreous length: 15.7 mm Optic nerve width: 4.4 mm. Eyeball morphology irregular, lens echo enhanced, uneven medium-level echoes; flocculent weak echoes in the vitreous. Normal morphology of both eyes, with no obvious abnormal echoes in the lenses or vitreous No obvious mass echoes were observed behind both eyeballs The left superior ophthalmic vein was significantly widened, with an inner diameter of 11 mm, low echo filling within the lumen, partial left eyeball compression CDFI showed no obvious blood flow signals within the lumen	Stable vital signs, left-eye blindness, no significant headache The patient was followed up in outpatient clinic for 1 year with no recurrence of CCF. No antiplatelet or anticoagulant drugs were used before or after the surgery
Case 2	6 months	5% residual shunt flow remained from the fistula to the contralateral intercavernous sinus	PRE: No pulsating proptosis was observed (clinically negative for CCF). Visual acuity in the right eye was 0.9. POST: visual acuity in the right eye was 1.0, and visual acuity in the left eye was normal	The right ear vascular murmur disappeared, and the patient had some residual headache, but the general condition improved. No antiplatelet or anticoagulant drugs were used before or after the surgery
Case 3	1 year	Reduction in cavernous sinus opacification. Cavernous sinus fistula resolved with a minimal residual leak. Preserved distal blood flow	PRE: Coma. The patient was admitted due to trauma, with rupture of the right eyeball. Before the interventional procedure, the right eye’s contents had already been enucleated POST: The patient was in a dazed state and unable to cooperate with vision testing. The patient was discharged after 1 month, with a visual acuity of 1.0 in the left eye	Although he remained in a coma for some time after the procedure, gradual neurological improvement was observed. No antiplatelet or anticoagulant drugs were used before or after the surgery. No recurrence of CCF.
Case 4	3 years	Satisfactory embolization of the traumatic left ICA–cavernous sinus fistula	PRE: left eye blindness POST: Visual acuity of the left eye: no light perception. Intraocular pressure: 31 mmHg. Eyeball was protruding, ocular movement restricted, conjunctiva congested and edematous protruding outside the globe, cornea was clear, anterior chamber deep, pupil slightly dilated and fixed, lens transparent, fundus not clearly visible	No antiplatelet or anticoagulant drugs were used postoperatively. Improved ability to open the left eye After 3 years, light perception in the left eye, no recurrence of left-sided CCF.
Case 5	8 months	significant reduction in the fistula flow	PRE: blurred vision in the right eye with preservation of distal arterial patency POST: the patient remained comatose and could not cooperate with vision testing	No recurrence of CCF. The patient was alert, with normal visual acuity and visual field No antiplatelet or anticoagulant drugs were used postoperatively
Case 6	1 year	Cavernous sinus fistula was completely occluded. Distal blood flow was preserved	PRE: right eye redness, pain, blurred vision, and diplopia. visual acuity was 0.15 in the right eye and 0.3 in the left eye. Intraocular pressure was 27.0 mmHg in the right eye and 19.5 mmHg in the left eye POST: round pupils of 3 mm diameter; normal light reflex. Visual acuity: right eye: 0.25, left eye: 0.3. Limited abduction of right eye	Clear vision and resolution of the pain and redness. Disappearance of double vision, dizziness, headaches, nausea, or vomiting. Normalization of appetite and sleep No antiplatelet or anticoagulant drugs were used postoperatively

A notable complication in the management of traumatic CCFs is the potential association with traumatic cerebral aneurysms (TCAs). TCAs can complicate the diagnosis and treatment of CCFs, as they may be difficult to detect before CCF occlusion due to masking effects from the parent artery or fistula drains. This was evident in several of the cases reported in this study, where a TCA could either complicate or alter the approach to endovascular intervention. Prompt detection and management of TCAs are critical to preventing fatal complications, such as spontaneous rupture (Thrishulamurthy et al., 2024).

Traumatic CCFs often manifest with a constellation of symptoms, including pulsatile exophthalmos, conjunctival chemosis, and cranial nerve deficits. These findings are consistent with previous studies that describe the varying clinical presentations of direct and indirect CCFs (Hu et al., 2022; Meyers et al., 2002). In this study, all patients exhibited ocular manifestations, underscoring the importance of early ophthalmologic and neurological assessment.

Traumatic CCFs, especially those involving bilateral presentations, pose additional diagnostic and therapeutic challenges. Bilateral post-traumatic CCFs can present with symptoms such as exophthalmos, diplopia, and ophthalmoplegia (Camara et al., 2024), which align with the clinical manifestations observed in the patients of this study. The management approach described by Camara et al. (Camara et al., 2024), involving transarterial embolization with coils, mirrors the strategy used in many of the cases in this series, underscoring the importance of timely intervention to achieve successful outcomes and prevent vision loss.

DSA remains the benchmark for diagnosing CCFs, providing high-resolution imaging necessary for treatment planning (Timol et al., 2018). In cases where the fistula anatomy was complex or difficult to access via standard arterial approaches, transvenous embolization through the superior ophthalmic vein or inferior petrosal sinus was utilized (Thiex et al., 2014; Han et al., 2019; Liang et al., 2024). The choice of access route was dictated by anatomical considerations and the extent of venous drainage involvement, as also recommended in prior literature (Korkmazer et al., 2013; Klisch et al., 2003). This aligns with the findings reported by Ma et al. (2023), where the combination of Onyx and coils was used in a cohort of patients, some of whom had traumatic CCFs. The use of transvenous and transarterial approaches is highlighted as a critical component of successful management, with the transvenous approach often being the first choice for indirect CCFs. The outcomes reported in this study, including high occlusion rates and symptom improvement, are in agreement with the results seen in this study, where most patients showed stabilization and symptom resolution after embolization.

Endovascular embolization has become the preferred treatment modality for CCFs, given its minimally invasive nature and high success rate (Plasencia and Santillan, 2012). Coil embolization was employed in all cases, often in conjunction with stent-assisted techniques to ensure complete occlusion of the fistula while preserving the parent artery. The use of embolic glue (EVAL-I in some cases of this study) and of similar type of n-butyl cyanoacrylate glue-like embolic materials in other selected cases (Ohlsson et al., 2016) demonstrated its utility in achieving durable occlusion, particularly in instances of persistent retrograde venous drainage. The necessity of combining multiple embolization materials highlights the complexity of managing high-flow fistulas, where single-modality treatment may be insufficient. The use of multimodal endovascular therapy, combining various embolization materials such as coils and Onyx, has also been shown to significantly improve occlusion rates (Alatzides et al., 2023). This approach, demonstrated in a large cohort of patients, further supports the strategy of using a tailored, multifaceted approach to tackle complex CCF cases.

The identification and targeted embolization of the “first pouch” play a pivotal role in the successful treatment of complex CCFs. Advanced imaging modalities such as Vaso-CT and 3D rotational angiography are valuable tools in delineating the fistulous architecture and flow dynamics, enabling precise localization and sizing of the initial venous compartment. In our cohort, we adopted a strategy of proximal flow reduction using coils, followed by ONYX embolization. This sequential approach allowed for controlled delivery of the embolic agent while minimizing the risk of distal venous migration, which could otherwise result in complications such as orbital compression or restricted ocular motility. Although ONYX-alone embolization is increasingly reported, particularly in complex cases, combining coils with ONYX may offer better hemodynamic control in high-flow fistulas. Furthermore, intraoperative awareness of trigemino-cardiac reflex and postoperative surveillance for cranial nerve palsies were integral to our perioperative management; no such complications were observed. These considerations support a tailored endovascular approach guided by detailed anatomical and functional assessment.

In cases where transarterial embolization was unsuccessful, a transvenous approach was considered. For instance, in Case 1, after the failure of transarterial treatment, an alternative venous route was explored. The ophthalmic vein approach proved particularly advantageous due to its proximity to the lesion and ease of catheterization, offering a viable access option for complex CCFs. This highlights the importance of individualized treatment strategies and the need for flexibility in endovascular intervention.

Despite successful embolization, some patients experienced persistent visual deficits, reflecting the irreversible ischemic damage sustained prior to intervention. This aligns with findings from previous studies that suggest delayed treatment is a significant factor contributing to poor visual outcomes (Alam et al., 2019; Gasparian and Chalam, 2021). Therefore, expediting diagnosis and treatment remains critical in optimizing patient recovery and reducing long-term morbidity.

Bilateral CCFs, as seen in one patient in this study, pose additional therapeutic challenges. These cases require careful hemodynamic assessment to ensure adequate collateral circulation before intervention. The staged embolization approach used in these cases aligns with current best practices for managing complex, high-flow arteriovenous shunts. The presence of associated traumatic brain injuries and systemic complications further complicates management, emphasizing the need for a collaborative approach involving neurosurgeons, interventional radiologists, and intensive care specialists.

The variability in postoperative outcomes underscores the necessity for personalized treatment strategies. While most patients achieved fistula closure and symptom stabilization, persistent neurological deficits in some cases highlight the limitations of current interventions. Future advancements in biomaterials for embolization, improved catheter navigation techniques, and refined imaging modalities may further enhance treatment precision and long-term success rates. Additionally, the role of adjunctive therapies, such as neuroprotective agents, warrants further exploration to mitigate ischemic damage and optimize patient recovery.

Long-term follow-up is essential in CCF management, as recurrence can occur despite initial successful embolization. In this series, follow-up angiography revealed stable occlusion in most patients, consistent with the reported high efficacy of endovascular interventions (Texakalidis et al., 2021; Korkmazer et al., 2013). However, subtle recanalization remains a concern, particularly in cases with residual low-flow shunting. Further studies are needed to establish standardized protocols for post-treatment surveillance and to determine the optimal duration of follow-up imaging.

## 5 Conclusion

The cases presented highlight the intricate nature of traumatic CCFs and the necessity of a tailored, multidisciplinary approach to management. The main novel therapeutic implication of this study lies in its exploration of embolization strategies for complex CCFs, specifically focusing on the optimal selection of techniques and materials that ensure dense, recurrence-free occlusion while minimizing the risk of mass effect caused by embolic agents. Endovascular embolization remains the primary treatment modality, with coil and glue embolization demonstrating high efficacy in achieving fistula closure. However, challenges such as residual shunting, complex anatomical variations, and associated trauma-related complications require continuous advancements in imaging and intervention strategies. Future research should focus on long-term outcomes, recurrence prevention, and optimizing patient-specific treatment protocols. These findings reinforce the critical role of early diagnosis, strategic intervention, and interdisciplinary expertise in managing traumatic CCFs and mitigating long-term morbidity.

## Data Availability

The original contributions presented in the study are included in the article/Supplementary Material, further inquiries can be directed to the corresponding author.
