# Supplementary material for: Multimodal endovascular management of traumatic carotid-cavernous fistulas: case series and lessons learned
Source: Front Pharmacol. 2025 May 30;16:1602971. doi: 10.3389/fphar.2025.1602971 (PMC12163331; doi:10.3389/fphar.2025.1602971)
Supplement: Supplementary file 1 [file Supplementaryfile1.docx]

**Supplemental Material**

**Multimodal Endovascular Management of Traumatic Carotid-Cavernous Fistulas: Case Series and Lessons Learned**

1. **SUMMARY OF THE 6 CASES.**

**Case 1. Management of Traumatic Left Carotid-Cavernous Fistula with Two-Stage Endovascular Intervention in a 38-Year-Old Male.**

*Diagnosis:* Traumatic Left Internal Carotid Artery (ICA) Cavernous Sinus Fistula (CCF) due to trauma.

*Clinical Findings:*

- Left eyelid mild edema, slight exophthalmos, slight ptosis, mild conjunctival congestion.
- No abnormalities in ocular movements.
- No scleral icterus.
- Lens opacities present.
- Left eye blind, right eye normal vision.
- Left pupil diameter approximately 4 mm, absent light reflex.
- Right pupil diameter about 3 mm, normal light reflex.
- Left eye auscultation: blowing sound synchronous with pulse.

*Imaging:*

- DSA (Digital Subtraction Angiography) on June 19, 2018 showed a left ICA cavernous sinus fistula with significant eye venous congestion, but the exact location of the fistula was unclear.

*First Surgery (June 22, 2018):*

- Under general anesthesia, the Seldinger technique was used to puncture the right femoral artery, and an 8F sheath was inserted.
- A 6F catheter was used to perform left ICA angiography, revealing a cavernous sinus AV fistula.
- The 6F Envoy guiding catheter was advanced to the cervical segment of the left ICA, and a microcatheter was used to probe the fistula's exact location.
- A series of embolization coils (14×30, 12×30, 10×30, 9×30) were used to occlude the fistula.
- A stent was placed for protection to prevent arterial damage during coil embolization.
- Post-embolization angiography showed satisfactory occlusion of the fistula with the contrast agent retention in the left eye venous system.

*Post-operative Imaging:*

- Angiography after the first procedure showed a complete occlusion of the fistula with good distal ICA blood flow.

*Second Surgery (August 2, 2018):*

- During the second procedure, angiography revealed coil embolization at the fistula site, but the fistula persisted with some retrograde flow to the eye veins.
- A second intervention via left external jugular vein access was attempted but unsuccessful.
- The eye venous approach was then used to successfully access and embolize the remaining fistula.

*Post-operative Status (August 14, 2018):*

- The patient was awake with stable vitals, left eye blindness, no significant headache, and normal right eye vision.

**Case 2. Endovascular Treatment of Right Carotid-Cavernous Fistula in a 31-Year-Old Female Following Trauma**

*Diagnosis:*

1. Right ICA Cavernous Sinus Fistula.
2. Craniocerebral trauma (brain contusion, subarachnoid hemorrhage, right temporal bone fracture, etc.).
3. Hyperuricemia.

*Clinical Findings:*

- Right eye conjunctival congestion and slight proptosis.
- Mild right ear tinnitus for 2 months and right eye ball congestion for 2 days.

*Surgical Procedure:*

- Under general anesthesia, right femoral artery access was achieved using the Seldinger technique.
- 5F catheter angiography revealed a right ICA cavernous sinus fistula, with arterial flow directed into the eye veins and opposite cavernous sinus.
- Fistula embolization was carried out using coils and glue, though the fistula persisted due to anatomical complexity.
- The fistula was partially occluded after multiple embolization attempts using coils and glue.

*Post-operative Outcome:*

- The right ear vascular murmur disappeared, and the patient had some residual headache, but the general condition improved.

**Case 3. Severe Head and Chest Trauma with Bilateral Carotid-Cavernous Fistula in 18-Year-Old Patient Following Fall.**

*Diagnosis:*

1. Severe open traumatic brain injury (including brain herniation, multiple contusions, and subdural hematoma).
2. Bilateral ICA Cavernous Sinus Fistulas.
3. Other trauma-related injuries (including pulmonary contusions, rib fractures, and a traumatic eye rupture).

*Clinical Findings:*

- The patient presented in a coma (GCS 5), with bilateral fixed pupils, and an open traumatic brain injury.

*Surgical Intervention:*

- Under general anesthesia, angiography revealed bilateral ICA cavernous sinus fistulas with significant arterial-to-venous shunting.
- Multiple coils and embolic agents were used to occlude the fistulas, with a final satisfactory result.

**Case 4. Traumatic Left Carotid-Cavernous Fistula with Left Eye Blindness in a 64-Year-Old Female: Successful Endovascular Treatment.**

*Diagnosis:*

- Traumatic Left ICA Cavernous Sinus Fistula.
- Left eye blindness.

*Surgical Procedure:*

- Angiography demonstrated a traumatic fistula at the left ICA cavernous sinus region.
- A microcatheter was used to perform coil embolization and glue injection, achieving a satisfactory occlusion of the fistula.

*Post-operative Outcome:*

- Left eye blindness persisted, but vision impairment improved slightly.

**Case 5. Ruptured Internal Carotid Artery Bifurcation Aneurysm with Subarachnoid Hemorrhage and Left Carotid-Cavernous Fistula: Complex Endovascular Management in a 64-Year-Old Female.**

*Diagnosis:*

- Ruptured aneurysm at the bifurcation of the left ICA with subarachnoid hemorrhage.
- Left ICA cavernous sinus fistula.

*Surgical Procedure:*

- After aneurysm embolization with stent-assisted coiling, a microcatheter was advanced through the left ICA to occlude the cavernous sinus fistula.
- Multiple embolization coils and glue injections were performed, with post-operative angiography showing a satisfactory occlusion of the fistula.

*Post-operative Status:*

- The patient was transferred to the intensive care unit for further management due to critical comorbidities.

**Case 6. Endovascular Treatment of Left Carotid-Cavernous Fistula with Adjacent Orbital and Ocular Manifestations in a 46-Year-Old Female**

*Diagnosis:*

- Left ICA cavernous sinus fistula.
- Paralytic strabismus, entropion, trichiasis, and keratitis in the right eye.

*Surgical Procedure:*

- Diagnostic angiography confirmed the fistula location and drainage sites.
- The interventional procedure included using multiple microcatheters and coils to occlude the fistula.
- Additional EVAL-I glue was injected to completely occlude the fistula, preserving distal blood flow.

*Post-operative Status:*

- The patient’s condition improved significantly, with resolution of eye redness, pain, double vision, and visual acuity.
- Post-procedure angiography confirmed successful occlusion of the fistula.
- No complications at the puncture site, and the patient was stable upon discharge.

.
